# Clinical effectiveness of an individually tailored strengthening programme, including progressive resistance exercises and advice, compared to usual care for ambulant adolescents with spastic cerebral palsy (ROBUST trial): a parallel group randomized controlled trial

**DOI:** 10.1302/2633-1462.65.BJO-2024-0268

**Published:** 2025-05-01

**Authors:** Sally Hopewell, David J. Keene, Ioana Marian, Daniel C. Perry, Ines Rombach, Morag Andrew, Catherine Barry, Loretta Davis, Gregory Firth, Heidi Fletcher, Beth Fordham, Vivi Gregory Osborne, Helen Gregory Osborne, Lesley Katchburian, Joanna O'Mahoney, Jeremy Parr, Rachel Rapson, Jennifer Ryan, Elnaz Saeedi, Megan Stone, Helen Wood, Tim Theologis

**Affiliations:** 1 Oxford Clinical Trials Research Unit, Nuffield Department of Orthopaedics, Rheumatology and Musculoskeletal Sciences, University of Oxford, Oxford, UK; 2 University of Exeter, Exeter, UK; 3 University of Liverpool, Liverpool, UK; 4 School of Medicine and Population Health, University of Sheffield, Sheffield, UK; 5 Newcastle upon Tyne NHS Foundation Trust; Newcastle University Population Health Sciences Institute, Newcastle upon Tyne, UK; 6 Surgical Interventions Trials Unit, Nuffield Department of Orthopaedics, Rheumatology and Musculoskeletal Sciences, University of Oxford, Oxford, UK; 7 Maidstone and Tunbridge Wells NHS Trust in Kent, Maidstone, UK; 8 Patient and Public Involvement Representative, London, UK; 9 Great Ormond Street Hospital for Children NHS Foundation Trust, London, UK; 10 Children and Family Health Devon, Torbay and South Devon NHS Foundation Trust, Torquay, UK; 11 CP-Life Research Centre, RCSI University of Medicine and Health Sciences, Dublin, Ireland; 12 Nuffield Department of Orthopaedics, Rheumatology and Musculoskeletal Sciences, University of Oxford, Oxford, UK

**Keywords:** Cerebral palsy, Strengthening exercises, Physiotherapy trial, resistance exercises, randomized controlled trial, Physiotherapists, physiotherapy, exercise programme, clinicians, muscle strength, Gross Motor Function Classification System, spastic cerebral palsy

## Abstract

**Aims:**

Muscle strengthening exercises are one of the interventions frequently prescribed by physiotherapists for adolescents with cerebral palsy (CP). However, there is wide variability in the exercise regimes used and limited evidence of their effectiveness. The ROBUST trial will assess the clinical effectiveness of an individually tailored strengthening programme, including progressive resistance exercises and advice, compared to usual care for ambulant adolescents with spastic CP.

**Methods:**

We are conducting a multicentre, two-arm, parallel group, superiority randomized controlled trial. We will recruit adolescents aged 12 to 18 years with a diagnosis of spastic CP (bilateral or unilateral) Gross Motor Function Classification System (GMFCS) levels I to III who are able to comply with the assessment procedures and exercise programme with or without support. Participants will be recruited from at least 12 UK NHS Trust physiotherapy and related services. Participants (n = 334) will be randomized (centralized computer-generated 1:1 allocation ratio) to either: 1) a progressive resistance exercise programme, with six one-to-one physiotherapy sessions over 16 weeks; or 2) usual NHS care, with a single physiotherapy session and an assessment session, and advice regarding self-management and exercise.

**Conclusion:**

The primary outcome is functional mobility measured using the child-/parent-reported Gait Outcomes Assessment List (GOAL) at six months. Secondary outcomes are: clinician-assessed muscle strength (Five Times Sit-to-Stand Test) and motor function (timed up and go test) at six months; functional mobility (GOAL) at 12 months; independence (GOAL subdomain A), balance (GOAL subdomain A, B, D), pain and discomfort (GOAL subdomain C), health-related quality of life (youth version of the EuroQol five-dimension questionnaire; EQ-5D-Y), educational attendance, exercise adherence, and additional physiotherapy treatment (six and 12 months). The primary analysis will be intention to treat.

Cite this article: *Bone Jt Open* 2025;6(5):517–527.

## Introduction

Cerebral palsy (CP) encompasses a group of permanent developmental disorders affecting movement and posture and causing activity limitation.^1^ CP affects approximately one in 400 children in the UK,^2^ and represents a lifetime disability with significant socioeconomic consequences. Functional mobility is best classified by the Gross Motor Function Classification System (GMFCS), an international standard based on the severity of the motor disability.^3^ About 65% of children with CP are ambulant, either with walking aids (GMFCS level III) or without (GMFCS levels I and II). CP is also classified according to the affected body areas (one side of the body (unilateral), both sides of the body (bilateral)), and the neurological pattern (spastic, dyskinetic, ataxic, mixed).^1^

In 70% of people, CP predominantly causes spasticity (increased muscle stretch reflex activity and passive stiffness). The increased muscle tone leads to progressive muscle stiffness and deficient longitudinal muscle growth.^4^ This then causes secondary joint contracture, bone deformity, and pain.^5^ In addition to the stiffness caused by spasticity, there is underlying muscle weakness, which contributes significantly to the motor function impairment.^6^ In adolescence, the increase in body mass challenges lower limb function as problems with muscle weakness become more evident. This leads to a decline in motor function, with an impact on activity and participation.^3^ Improving or maintaining strength of lower limb muscles is therefore important in adolescence to minimize functional decline.^7^

Physiotherapy is introduced early in the treatment of children and young people with CP to support motor development and prevent musculoskeletal problems.^8^ Physiotherapy provision throughout childhood represents a substantial time and cost burden for the child, family, and NHS. The optimization of physiotherapy provision for children and young people with CP was identified as a top priority in the British Academy of Childhood Disability (BACD) James Lind Alliance Childhood Disability Priorities Setting Partnership.^9^ Research on the effectiveness of physiotherapy in preventing deformity and the need for surgery was also prioritized by the British Society for Children’s Orthopaedic Surgery James Lind Alliance Paediatric Orthopaedic Surgery Priorities Setting Partnership.^10^ A 2018 scoping review also highlighted the need for evidence-based physiotherapy interventions in children and young people with CP, which are deliverable through the NHS and focus on improving activity and participation in a child- and family-friendly manner.^11^

Muscle strengthening exercises are often used by the physiotherapists treating older children and adolescents with CP.^12^ However, there is wide variability in the strengthening exercise programmes used, and the regimens are primarily based on guidelines for people without CP.^13^ A Cochrane review of exercise interventions for CP found low-quality evidence that resistance training may improve muscle strength, but does not improve motor function, gait speed, or participation in the short or intermediate term. However, all of the trials were small, resulting in considerable uncertainty; large, high-quality randomized trials were recommended.^14^ A 2019 systematic review showed some evidence that resistance training improved motor function in children with CP; however, again, the trials were small and heterogeneous in the type of exercise programme and choice of comparator.^15^

The type of strengthening exercises used in the above trials ranged from resistance training to multijoint body weight or weight-loaded functional exercises (e.g. sit-to-stand, lunging, step-ups, side-stepping, squatting).^14^ Settings included the home, clinic, or educational setting, and duration of the training periods varied between four and 20 weeks. Most published interventions were delivered with the frequency of three sessions per week. Programmes were individually tailored, based either on adjusting weight loading according to body weight or on the individual’s ability to undertake a predefined number of repetitions. Most studies included gradual progression of the programme to increase weight loading and/or number of repetitions. The STAR trial, published most recently in 2020,^16^ evaluated the effect of a 30-session (ten supervised and 20 unsupervised home-based) resistance training programme compared to usual care in adolescents with CP and found no difference in gait efficiency, activity, and participation. However, again, this trial was small, and the exercises included in this programme only targeted one specific muscle group.

None of the previous studies on strengthening exercises have included a behaviour change component. A strengthening intervention can only be effective if the target population performs and maintains the proposed exercise behaviours. There is evidence to suggest that the addition of behaviour change components to exercise interventions increases the likelihood that the target population will perform the prescribed exercises.^17^ The COM-B change provides a theoretically based framework for designing complex interventions to enhance behaviour change.^18^

Given the resources, time, and effort for young people, parents, and professionals required to deliver strengthening regimes, there is a pressing need to evaluate clinical effectiveness.^9,11^ The literature supports testing a clearly defined strengthening intervention that is acceptable to young people and families, widely supported by physiotherapists and deliverable in the NHS. As highlighted by the UK’s National Institute for Health and Care Excellence (NICE) guidance on management of spasticity in young people,^19^ the intervention should be adolescent-centred and focused on activity and participation goals.^11^ The burden on the young person and family should be minimized, and delivery of the intervention should be as unobtrusive as possible.

We describe a randomized controlled trial, using a parallel group design, to assess the effectiveness of an individually tailored strengthening programme, underpinned by evidence-based behaviour change theory, compared to usual care among ambulant adolescents with spastic CP. The strengthening intervention has been co-designed with children and young people with CP, their parents, and healthcare professionals to maximize deliverability within the context of routine care.

The primary objective is to determine whether an individually tailored 16-week strengthening programme, including progressive resistance exercises and advice, improves functional mobility at six months (measured using the child-/parent-reported Gait Outcomes Assessment List (GOAL) questionnaire),^20^ compared to usual care among ambulant adolescents with spastic CP.

Secondary objectives are to investigate whether there is any difference between the two groups at six months in clinician-assessed muscle strength (Five-Time Sit-to-Stand test)^21^ and motor function (Timed Up and Go test);^22^ at 12 months, in functional mobility (GOAL questionnaire);^20^ at six and 12 months, in independence (GOAL subdomain A); balance (GOAL subdomain A, B, D), pain and discomfort (GOAL subdomain C), health-related quality of life (youth version of the EuroQol five-dimension questionnaire (EQ-5D-Y)),^23^ educational attendance, exercise adherence, and additional physiotherapy treatment.

## Methods

### Study design

**Fig. 1:**
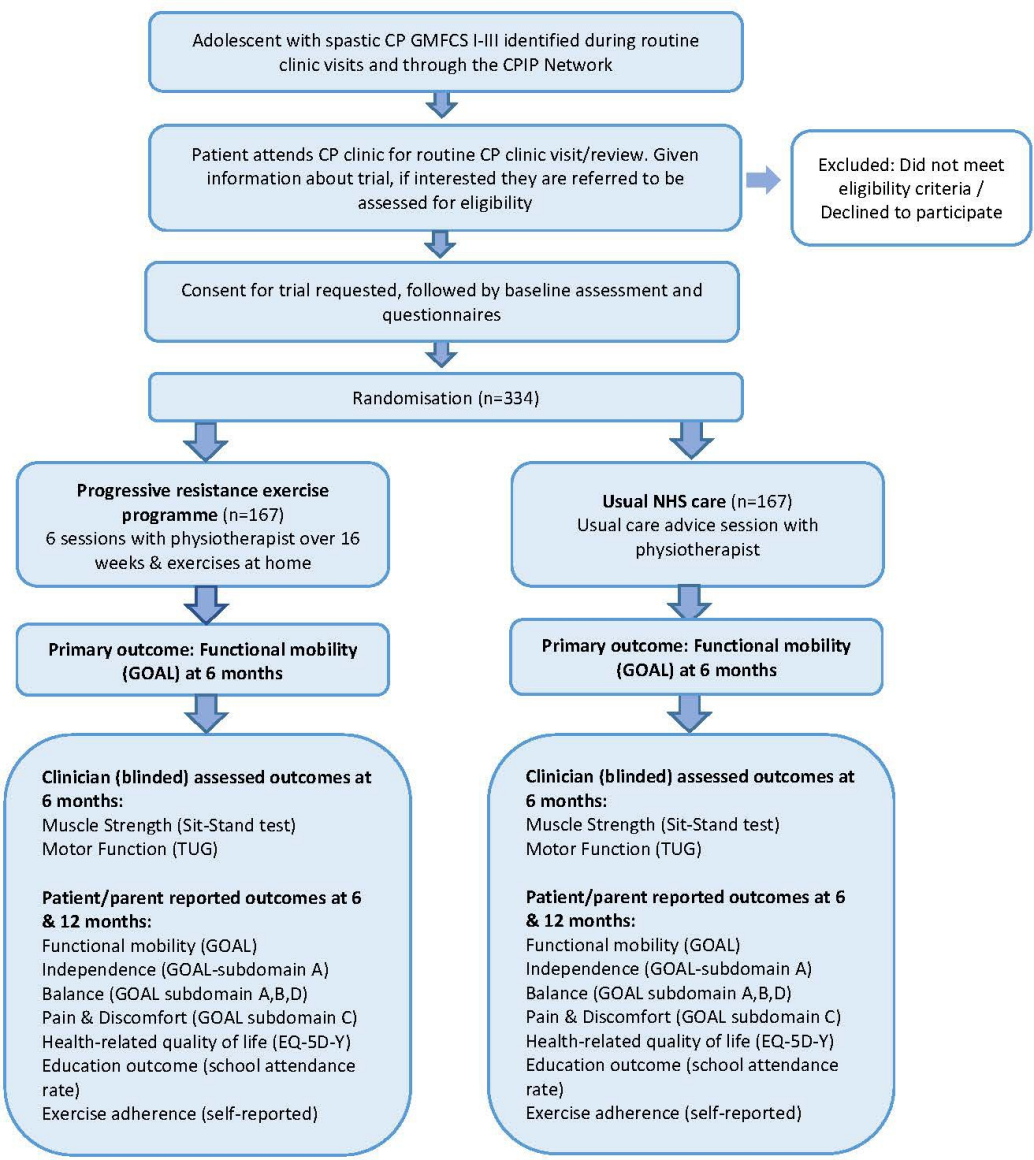
Study flow diagram for ROBUST trial. CP, cerebral palsy; CPIP, cerebral palsy integrated pathway; EQ-5D-Y, child-friendly version of the EuroQol five-dimension questionnaire; GMFCS, Gross Motor Function Classification System; GOAL, Gait Outcomes Assessment List; TUG, Timed Up and Go test.

The ROBUST trial is a multicentre, two-arm, parallel group, superiority, randomized controlled trial with an embedded internal pilot (Figure 1). ROBUST will be conducted alongside the SPELL trial, which will assess whether a child-specific dynamic stretching programme, overseen by a physiotherapist over 16 weeks, improves functional mobility (measured using the child-/parent-reported GOAL questionnaire) at six months in ambulant children aged four to 11 years (i.e. from their fourth to the day before their 12th birthday), with spastic CP compared to usual care. The SPELL trial protocol is published separately.^24^

### Setting

Participants will be identified and recruited from at least 12 UK NHS Trusts, providing paediatric physiotherapy services to children and young people with CP. Sites will be hospital- or community-based, depending on the configuration of local services.

### Recruitment of participants, screening, and eligibility assessment

The ROBUST trial will recruit adolescents aged 12 to 18 years (i.e. from their 12th to their 18th birthday) with a diagnosis of predominantly spastic CP (bilateral or unilateral), which is graded as GMFCS levels I, II, or III, who consent to their community physiotherapy service and GP to be informed of their participation in the trial. Participants should be able to complete the assessment procedures and the exercise programme with or without support from their carer.

Patients will be excluded from participation in this study if they had orthopaedic surgery of the lower limbs or selective dorsal rhizotomy in the past 12 months, or planned (i.e. date confirmed) in the next six months; if they had lower limb botulinum toxin injections or serial casting in the past four months, or planned (i.e. date confirmed) in the next six months; if they are regularly performing a structured resistance exercise programme focused on resistance training as part of their usual physiotherapy routine; and/or if they are unable to comply with the assessment procedures and exercise programme with or without support.

Adolescents with a diagnosis of spastic CP (GMFCS levels I to III)^3^ and who meet current indications for physiotherapy will be screened for eligibility. Sites will identify potential participants through the Cerebral Palsy Integrated Pathway (CPIP) database.^25^ The CPIP database is a national database for the assessment of the musculoskeletal system, including hip surveillance in children and adolescents with CP. All children with CP are offered an annual CPIP musculoskeletal assessment by a community physiotherapist. Not all people with CP attend hospital; therefore, CPIP offers a unique opportunity to identify children with CP in the community, particularly in underserved areas where access to hospital-based services may be challenging. Not all hospitals are part of the national CPIP database, so potential participants will also be identified and recruited through routine paediatric, orthopaedic, and physiotherapy healthcare visits.

If eligible, adolescents and their parents will be provided with information about the trial, including an ‘explainer video’, age-appropriate participant information sheet (i.e. 12- to 15-year-olds, 16- to 18-year-olds parent/guardian or consultee participant information sheet), and a verbal explanation of the trial and trial procedures, and asked if they wish to be considered for the trial. Those meeting the eligibility criteria and wishing to participate in the trial will then be approached for informed consent. Participants who do not meet the eligibility criteria, or who do not wish to participate, will continue to receive their standard NHS physiotherapy treatment. We will record anonymous information, via site screening logs, on the age, sex, ethnicity, and social deprivation index of those who decline to participate so that we can assess the generalizability of those recruited. The reasons for any potential participants declining to be part of the study will also be recorded.

### Informed consent and baseline assessment

After participants have been assessed for eligibility, informed consent for participation in the trial will be sought by a physiotherapist or other healthcare professional trained in Good Clinical Practice. They will explain the details of the trial and ensure that the potential participant and their parent/guardian has sufficient time to consider whether or not to participate. Informed consent will be obtained in line with NHS Health Research Authority guidance for research involving children (Supplementary Material).^26^ Informed consent will usually be obtained electronically in clinic with the consent/assent form being completed directly on the trial database (REDCap).^27,28^ Where appropriate, e-consent may also be obtained remotely, following an initial contact by a member of the site trial team to introduce the study to the participant and their parent/guardian.

Participants with the support of their parent/guardian will then be asked to complete the baseline assessment questionnaire that will record simple demographic information and baseline measurements for the primary and secondary outcomes (Table I and Table II). Clinician-assessed outcomes (i.e. muscle strength and motor function) at baseline will be recorded electronically by a physiotherapist at site.

**Table I.** Timepoints at which outcomes will be assessed.

Outcome	Measurement	Timepoint(s), mths
**Primary outcome**		
Functional mobility	Gait Outcomes Assessment List (GOAL) questionnaire^20^	0, 6, 12
**Secondary outcomes**		
Muscle strength (clinician assessed)	Five Times Sit-to-Stand Test for adolescents with CP^21^	0, 6
Motor function (clinician assessed)	Timed Up and Go test^22^	0, 6
Independence	GOAL subdomain A^20^	0, 6, 12
Balance	GOAL subdomains A,B,D^20^	0, 6, 12
Pain and discomfort	GOAL subdomain C^20^	0, 6, 12
Health-related quality of life	EQ-5D-Y^23^	0, 6, 12
Educational outcomes	Educational attendance record (days)	0, 6, 12
Exercise adherence	Participant/Parent self-reported adherence	6, 12
Additional physiotherapy treatment	Participant/Parent self-reported treatment	6, 12

CP, cerebral palsy; EQ-5D-Y, youth version of the EuroQol five-dimension questionnaire.

**Table II.** Participant timeline.

Timepoint (from randomization)	Pre-randomization	Baseline	0 to 4 mths	6-mth follow-up	12-mth follow-up
**Enrolment**					
Screening log	✓				
Eligibility confirmed	✓				
Informed consent	✓				
Randomization		✓			
**Interventions**					
Progressive resistance exercise programme (if randomized to)			✓*		
NHS usual care (if randomized to)			✓*		
**Assessments**					
Baseline demographic questionnaire	✓				
Clinician-assessed outcomes (muscle strength and motor function)	✓*			✓*	
Participant-assessed outcomes (questionnaire)	✓			✓	✓
Follow-up reminders				✓	✓

* Timepoints that require clinic/hospital attendance, but other assessments at these timepoints could be undertaken electronically/over the telephone.

### Randomization

Consented participants will be randomized (1:1) to either the intervention or control group using the REDCap randomization system (a centralized validated computer randomization programme). Randomization will be performed using a minimization algorithm (including a random element) to ensure balance between the two groups using the following minimization factors: centre, sex, anatomical CP distribution (bilateral vs unilateral), and GMFCS (levels I and II compared with III).

### Blinding

Participants will be informed of their treatment group by the research facilitator at the initial appointment. Participants will not be blinded to the treatment allocation, nor will the physiotherapists delivering the intervention. The trial statistician and data entry personnel will also not be blinded to the treatment allocation. Physiotherapists performing the six-month follow-up outcome assessment will be blinded to treatment allocation, where possible provided that staffing levels allow, as will the remaining members of the central trial management team until after the data analysis is complete.

### Interventions

Full details of the ROBUST trial interventions and their development will be published elsewhere in accordance with the TIDieR Statement;^29^ they are described here in brief.

Progressive resistance exercise programme: the participants randomized to the progressive resistance exercise programme will receive a 16-week individually tailored, structured exercise and advice programme, overseen by a physiotherapist, which consists of six one-to-one sessions. The 16-week training period allows time for the neurophysiological response to resistance training and for regular performance of exercises to become part of daily routine.^30^ The six physiotherapy-supervised sessions with the young person and the provision of parent/guardian training aim to initiate engagement in longer-term independent exercise. The first physiotherapy session will be up to 90 minutes, followed by five additional physiotherapy sessions of up to 60 minutes. The sessions will be offered at times that minimize disruption to education, consistent with NHS care for this patient group. Appointments will be coordinated so that participants typically start their first exercise session within two to four weeks of randomization, as per local appointment availability. Sessions will be in an outpatient setting or in the participants’ home or educational setting according to clinical need and local service provision. Participants will be advised to complete the exercise programme at home three times per week on non-consecutive days.

The programme follows the principles of progression for resistance exercises. The participant and physiotherapist will jointly choose up to five exercise options based on the functional goals identified during the assessment. These will be based on the participant functional mobility level (GMFCS I to II and III), while ensuring the exercise progression principles are consistent and monitored carefully. The development of the ROBUST library of progressive resistance exercises will be published elsewhere. The library provides a variety of exercises, accommodating different motor function levels and impairments, to allow for an individually tailored programme by selecting those most appropriate to a participant. The library is structured by grouping the exercises under the main target muscle group and allows for progression, ideally within the same muscle groups throughout the programme. Setting exercise intensity and load will be facilitated by use of the modified Borg scale of perceived exertion, an 11-point version of the Rating of Perceived Exertion (RPE)^31^ scale validated for quantifying the intensity of resistance exercise.^32^ Weighted vests and resistance exercise bands will be used to enable adequate loading without relying on expensive gym-based equipment. The number and type of exercises will be recorded using treatment logs by the trial physiotherapists. The volume of physiotherapy supervision is broadly consistent with routine practice among children with CP and existing NHS commissioning paradigms.^19^ Importantly, the intervention has been designed to ensure deliverability within an NHS setting.

To support adherence to the progressive resistance exercise programme, participants will have access to written instructions on the progressive resistance exercises chosen, including photos of each exercise and video instructions of the progressive resistance exercises chosen via the ROBUST study intervention website.^33^ To ensure accessibility, if families do not have ready access to a mobile device at home, then a device will be loaned to participants to enable them to use the digital intervention during the supervised exercise period. The intervention design and long-term behaviour change implementation have been underpinned by the capability-opportunity-motivation model of behaviour (COM-B) change for intervention development.^18^ The programme includes goal-setting and exercise diaries accessed via the intervention website, with joint problem-solving, monitoring, and motivation from the physiotherapist. The goal-setting and exercise diaries are for use between the participant and their physiotherapist, and will be reviewed at each physiotherapy session.

Usual NHS care: adolescents randomized to usual care will attend for a single session with a physiotherapist for an assessment, lasting up to 90 minutes. This will be in addition to physiotherapy appointments that they receive through their routine clinical care. Appointments will be coordinated so that participants typically receive their assessment session within two to four weeks of randomization, as per local appointment availability. To avoid contamination, where possible, physiotherapists delivering usual care will be different to those delivering the progressive resistance exercise programme. Participants and their parent/carer will be provided with NHS advice on self-management, including a participant information booklet on exercise and activity for young people with CP and continuation of any usual fitness/physical activity programme (as applicable).^19^

Participants allocated to the usual care group will not have access to the progressive resistance exercise programme. Usual care will be recorded using a treatment log maintained on the REDCap trial website by the trial physiotherapists. A guideline on what is considered ‘usual NHS care’ will be provided to the physiotherapists delivering it, and they will be trained to understand the components of this, to ensure they know the boundary of provision.

Concomitant care: all participants will be advised to maintain their usual physiotherapy care, which may include the use of orthotics, and other forms of treatment during the trial (as long as this does not include a progressive resistance exercise programme), but will be informed that they should use the usual clinical referral routes to do so. We will record and monitor any additional physiotherapy received outside of the trial intervention and prescribed during the trial follow-up period.

Training and monitoring of intervention delivery, adherence, and fidelity: physiotherapists delivering the trial interventions will have access to a comprehensive intervention manual and will be required to have undertaken trial-specific training, delivered by a research physiotherapist. The physiotherapists will be experienced practitioners, under the supervision of one of the research physiotherapists (CB, HW) on the central trial team. The training will include comprehensive guidance on the theory and practical delivery of the trial interventions.

A rigorous quality control programme will be conducted to ensure protocol and intervention fidelity (i.e. the exercises being undertaken according to the protocol). Quality assurance checks will be made by the trial team, who will observe treatment sessions by physiotherapists. Site visits will be conducted periodically (minimum one visit per site per year) to observe the recruitment, consent, and randomization procedures, data collection, follow-up assessments, intervention, and usual care session(s).

We will also monitor adherence to treatment (participants undertaking the prescribed number of sessions and exercises), by logging aspects of the intervention. This will include the name of the exercises prescribed, the duration of physiotherapy appointments attended (and any additional contact), the number of sessions per week undertaken at home without physiotherapy supervision, and whether the session was completed, partially completed, or not completed. Treatment logs will be maintained on the REDCap trial website by the trial physiotherapists. At six and 12 months of follow-up, we will also record longer-term self-reported adherence.

### Outcome measures

The primary outcome is functional mobility at six months measured using the patient-/parent-reported GOAL questionnaire.^20^ The GOAL is validated specifically for use in ambulant CP and is internationally accepted as the appropriate functional outcome measure for lower limb interventions in this population.^20^ The GOAL questionnaire consists of 48 items grouped into seven domains – A: activities of daily living and independence; B: gait function and mobility; C: pain, discomfort, and fatigue; D: physical activities, sports, and recreation; E: gait pattern and appearance; F: use of braces and mobility aids; and G: body image and self-esteem. A total GOAL score will be calculated in line with the scoring manual, ranging from 0 to 100, with higher values indicating better outcomes.

We will use the child version of the GOAL whenever possible and the parent version one if not. The families will be asked to decide which version is most appropriate as part of the consent process, and their decision will be recorded on the baseline clinical assessment form to enable consistent use of the same version during follow-up.

Secondary outcomes (Table I) are:

- Patient-/parent-reported outcomes at six and 12 months: independence measured using the GOAL subdomain A; balance measured using the GOAL subdomains A, B, D; pain and discomfort measured using the GOAL subdomain C;^20^ health-quality of life measured using the EQ-5D-Y;^23^ educational attendance record (number of days off school); exercise adherence; and additional physiotherapy treatment received outside of the trial.
- Clinician-assessed outcomes at six months: muscle strength measured using the Five Times Sit-to-Stand Test for adolescents with CP; and motor function using the Timed Up and Go test.^21,22^

The participants’ timeline through the trial is presented in Table II. A flowchart is presented in Figure 1.

### Patient and public involvement

Young people and their families have been involved in the development of this trial in a number of ways, including the format of the intervention and choice of primary outcome, and are representatives on the ROBUST Trial management group and trial steering group. We have also set up young person and parent advisory groups (YP/PAGs) to further support the trial and advise on the trial intervention materials, including the trial website, ROBUST Intervention app, participant and parent information sheets, and potential strategies to enhance recruitment and retention. In accordance with the Generation R advice (a network of young people supporting design of paediatric research in the UK),^34^ we will work with young people and parents on optimal ways to communicate the results of the trial to patients and the wider public.

### Adverse events

The potential occurrence of adverse events related to the intervention (Supplementary Material) will be recorded. The intervention has been designed to introduce a gradual increase in strength, thus minimizing the risk of musculoskeletal injury. Participants and their parent/guardian will be provided with information on the potential adverse events resulting from exercise as part of their treatment, including what they should do if they experience an adverse event, as would happen as part of standard NHS procedures. The participants and their parent/guardian will be asked to notify the treating therapist, as would occur during normal practice, if they suspect that they are suffering an adverse effect. In addition, at the six-month clinical follow-up visit, participants and their parent/guardian will be asked if they have experienced any adverse events. Expected general side effects of any form of exercise, such as delayed onset muscle soreness and temporary increases in pain (< seven days), will not be recorded as adverse events. Serious adverse events (SAEs) (defined as any unexpected medical occurrence that results in death, is life-threatening, requires hospitalization or prolongation of existing hospitalization, results in persistent or significant disability or incapacity, or is otherwise considered medically significant by the investigator) are very rare and are highly unlikely to occur as a result of the intervention delivered in this trial. However, if a SAE arises from the participant’s enrolment on the trial to their allocated intervention, standard procedures for recording and reporting SAEs will then apply.

### Follow-up data collection

Detail of the outcomes to be assessed, how they will be measured, and at which timepoints are shown in Table I. Patient-reported outcomes will be assessed using an electronic (online) questionnaire at six and 12 months from initial randomization. If requested, a paper-based version of the electronic questionnaire will be provided. For those who do not respond to the initial questionnaire, a reminder will be sent two weeks later. Telephone and email follow-up will be used, as applicable, to contact participants, and/or their parent/guardian, who do not respond to either the initial or reminder questionnaire. Clinician-assessed outcomes will be assessed at a face-to-face clinic appointment at six months by a blinded physiotherapist, where possible. Participants who do not attend this face-to-face clinic appointment will be contacted by phone by the local site team and a new clinic appointment sent.

### Data management

All data will be processed according to the General Data Protection Regulation (GDPR) and Data Protection Act 2018,^35^ and all documents will be stored safely in confidential conditions. All electronic patient-identifiable information will be held on a secure, password-protected database accessible only to authorized personnel. Paper forms with patient-identifiable information will be held in secure, locked filing cabinets within a restricted area. The processing of the personal data of participants will be minimized wherever possible by the use of a unique participant trial number on trial documents and any electronic databases. Access to participants' personal identifiable data will be restricted to individuals authorized to have access where this is necessary for their role.

### Sample size

The target sample size for the trial is 334 randomized participants (167 in each treatment group) (power analysis and sample size (PASS) v. 13). This will allow detection of a clinically meaningful moderate standardized effect size of 0.4 with a two-sided 5% significance level, 90% power, and allowing for 20% loss to follow-up. The standardized effect size of 0.4 corresponds to a difference of 6.8 points on the GOAL outcome measure,^20^ which ranges from 0 to 100, with a SD of 17. A difference of 6.8 is considered functionally important and achievable by key stakeholders, including patients who provided input in focus groups, and clinicians we surveyed in preparation for the trial. SDs of this magnitude have been reported in similar patient populations.^20,36^ The sample size assumptions will be reviewed after approximately 50% of the participants have been recruited.

### Statistical analysis

The primary analysis will use the randomized (intention-to-treat (ITT)) population, analyzing participants with available outcome data in their randomized groups, regardless of adherence to their allocated intervention. Primary and secondary outcome analyses will use two-sided 5% significance and 95% CIs with associated p-values.

Differences in GOAL scores between the trial groups will be estimated using a multilevel mixed effects regression model, allowing for repeated measures clustered within participants and for potential clustering within randomizing sites. The model will be adjusted for minimization factors (sex (male, female), anatomical disease distribution (bilateral compared with unilateral CP), and GMFCS level (levels I and II compared with III)), and other important prognostic factors (i.e. neurological pattern, epilepsy, or visual impairment), including the baseline GOAL scores. A treatment by time interaction will be included, indicating the protocol stipulated follow-up time to which the assessment refers. Model diagnostics, including approximate normality of the residuals, will be assessed. Adjusted mean differences and unadjusted mean differences between groups will be presented together with 95% CIs.

We will explore the effect of non-adherence with the randomized interventions using complier-average causal effect (CACE) analyses. Adherence will be defined as having completed all six physiotherapy sessions. However, the treating physiotherapist may decide that treatment is complete over a smaller number of sessions depending on the progress of the adolescent. Secondary outcomes will be analyzed using generalized linear models, with model adjustment as described for the primary analysis above. Subgroup analysis using predefined subgroups will investigate potential outcome predictors such as sex, anatomical distribution of the condition, GMFCS level, or other baseline characteristics. A statistical analysis plan (SAP) with full details of all analyses planned for the study data will be drafted early in the trial and finalized prior to any primary outcome analysis.

### Missing data

Missing data will be reported and summarized by group. The distribution of missing primary outcome data will be explored to assess the assumption of data missing at random. The potential impact of informative missing data (missing not at random) on the treatment effect will be investigated.

## Ethics and dissemination

Ethical approval was obtained from the South Central – Hampshire A Research Ethics Committee (REC: 23/SC/0231) and prospectively registered (ISRCTN 68282588. A data safety and monitoring committee has been appointed to independently review data on safety, protocol adherence, and recruitment in accordance with the Data Monitoring Committees: Lessons, Ethics, Statistics (DAMOCLES) charter.^37^ Direct access to research data will be granted to authorized representatives of the sponsor (University of Oxford, UK), regulatory authorities, or the host institution for monitoring and/or auditing of the trial to ensure compliance with regulations. Summary results will be included on the ISRCTN database within six months of the end of the trial. Requests for data (anonymized trial participant level data) will be provided at the end of the trial to external researchers who provide a methodologically sound proposal to the trial team (and who will be required to sign a data sharing access agreement with the sponsor) and in accordance with the National Institute for Health and Care Research (NIHR) guidance.

Trial findings will inform clinical practice for the management of ambulant adolescents with spastic CP and its results will be published in a high-impact open-access journal, in accordance with the NIHR’s policy on open-access research. The trial results will be reported following the CONSORT guidelines.^38^ We will use the TIDieR Statement for reporting the intervention,^29^ ensuring that replication is possible. Trial materials, including the physiotherapist training materials and intervention materials, will be made freely available via the trial website. Participants and their parent/guardian will be asked if and how they would like to be informed of the results as part of the consent process.

## Strengths and limitations

The ROBUST trial is a large multicentre RCT based across UK NHS hospital and community paediatric physiotherapy services. Using a two-arm, parallel group, pragmatic design, we will test the effectiveness of an individually tailored strengthening programme, including progressive resistance exercises and advice, overseen by a physiotherapist compared to usual NHS care in ambulant adolescents with spastic CP. The intervention has been designed to ensure deliverability within the NHS setting. Young people and their families were involved in the trial development, including development of the intervention, the creation of the trial materials, and choice of the primary outcome. It is not possible to blind the study participants or physiotherapists delivering the intervention.

**Take home message**

- Muscle strengthening exercises are one of the interventions frequently prescribed by physiotherapists for adolescents with cerebral palsy (CP).

- However, there is wide variability in the exercise regimes used and limited evidence of their effectiveness.

- This article describes the protocol for the ROBUST trial, which is a multicentre trial looking at the clinical effectiveness of an individually tailored strengthening programme, including progressive resistance exercises and advice, compared to usual care for ambulant adolescents with spastic CP.

## Data Availability

The data that support the findings for this study are available to other researchers from the corresponding author upon reasonable request.
